# Supplementary figures and images for: Urban Particulate Matter Enhances ROS/IL-6/COX-II Production by Inhibiting MicroRNA-137 in Synovial Fibroblast of Rheumatoid Arthritis
Source: Cells. 2020 Jun 2;9(6):1378. doi: 10.3390/cells9061378 (PMC7348867; doi:10.3390/cells9061378)

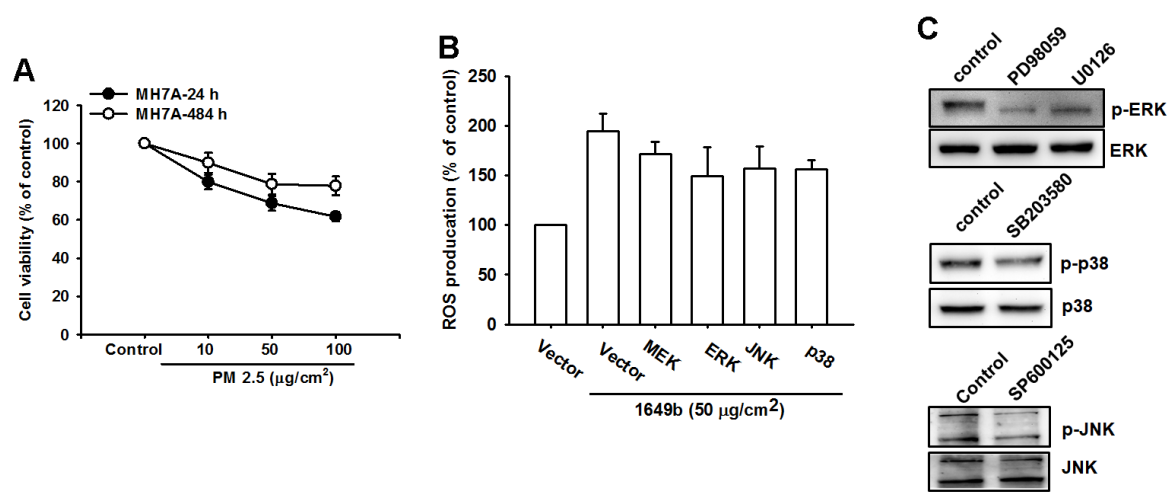

Figure S1.

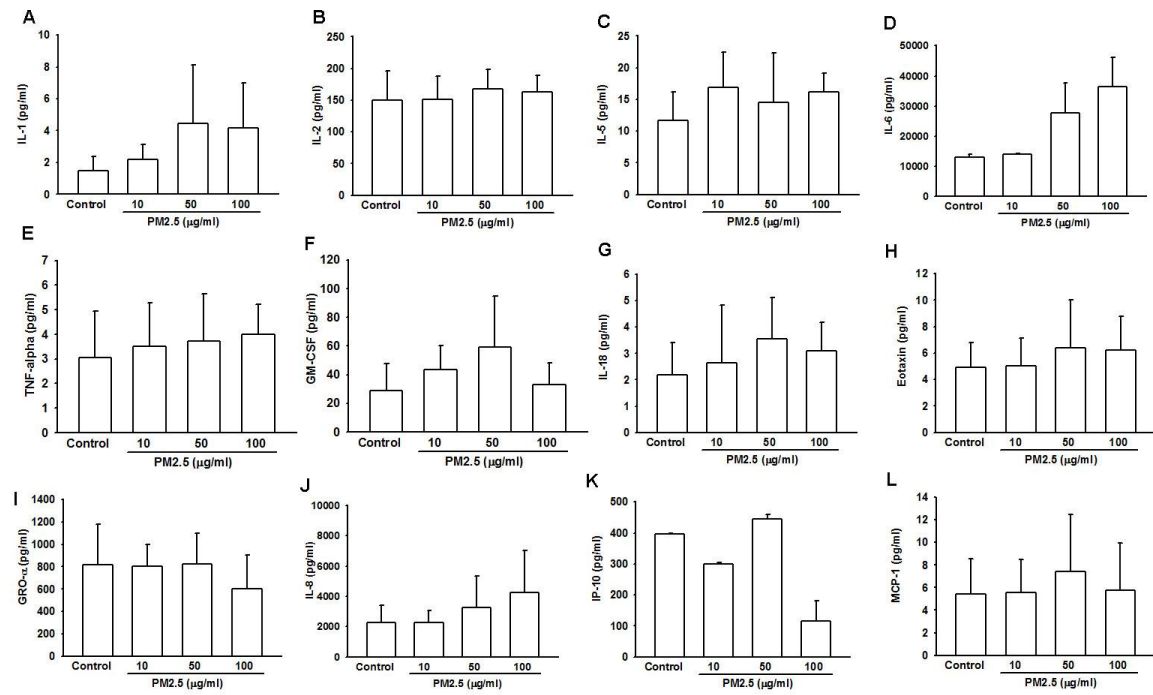

Figure S2

Supplement: Supplementary file 1 [file cells-09-01378-s001.pdf]
